# Sequence and Evolutionary Features for the Alternatively Spliced Exons of Eukaryotic Genes

**DOI:** 10.3390/ijms20153834

**Published:** 2019-08-06

**Authors:** Shi-Yi Chen, Cao Li, Xianbo Jia, Song-Jia Lai

**Affiliations:** Farm Animal Genetic Resources Exploration and Innovation Key Laboratory of Sichuan Province, Sichuan Agricultural University, Chengdu 611130, China

**Keywords:** eukaryotic genes, alternative splicing, exon features

## Abstract

Alternative splicing of pre-mRNAs is a crucial mechanism for maintaining protein diversity in eukaryotes without requiring a considerable increase of genes in the number. Due to rapid advances in high-throughput sequencing technologies and computational algorithms, it is anticipated that alternative splicing events will be more intensively studied to address different kinds of biological questions. The occurrences of alternative splicing mean that all exons could be classified to be either constitutively or alternatively spliced depending on whether they are virtually included into all mature mRNAs. From an evolutionary point of view, therefore, the alternatively spliced exons would have been associated with distinctive biological characteristics in comparison with constitutively spliced exons. In this paper, we first outline the representative types of alternative splicing events and exon classification, and then review sequence and evolutionary features for the alternatively spliced exons. The main purpose is to facilitate understanding of the biological implications of alternative splicing in eukaryotes. This knowledge is also helpful to establish computational approaches for predicting the splicing pattern of exons.

## 1. Introduction

It is well acknowledged that phenotypic and functional diversity are contributed by the variable transcription of genes in eukaryotes to a considerable extent [1]. The genic transcription could vary in terms of mRNA molecules and expression levels. In the case of the former, the same gene could be alternatively transcripted into more than one of the mRNA molecules with similar but not identical functions, and this one-to-several relationship of gene to mRNAs mainly results from alternative splicing of pre-mRNA [2]. Beside the well-known roles of regulating individual development and driving species evolution, increasing evidence also supports the supposition that disturbances of alternative splicing can be the causes or consequences of many diseases in humans [3]. Furthermore, economically important traits, such as reproduction, disease resistance and environmental fitness, have been successfully explained by alternative splicing events in animals and plants [4,5]. Therefore, the evolutionary dynamics and regulatory mechanisms for alternative splicing of eukaryotic pre-mRNAs have received considerable attention during the past decade [6,7,8].

In earlier studies, detection of alternative splicing events mainly depended on the expressed sequence tags (ESTs) with an estimation of about 50% of human genes subjected to alternative splicing [9]. With the aid of high-throughput RNA sequencing (RNA-Seq), two pioneering studies comprehensively investigated the transcriptome complexity in human and suggested that almost 95% of multiexon genes undergo alternative splicing [10,11]. The recent study also revealed that noncoding exons are universally alternatively spliced [7]. It seems reasonable to expect, therefore, that almost all multiexon genes would have the potential to be alternatively spliced and translated into multiple protein isoforms. However, this landscape may be challenged by the recent observation that most human genes actually have only a single main protein isoform [12]. Furthermore, computational analyses of alternative splicing events have been largely facilitated by the sophisticated bioinformatic tools during the past years, including the reference genome-guided and *de novo* approaches [13,14]. Due to the application of full-length transcript sequencing [15], such as by Pacific Biosciences (PacBio) and Oxford Nanopore Technologies (ONT), the accuracy, robustness and reliability have been largely improved for detecting alternative splicing events. Using the PacBio RS II platform, our lab successfully explored a large number of novel alternative splicing events in rabbits, a non-model organism [16].

A direct consequence of alternative pre-mRNA splicing is that all exons can be classified to be either constitutively or alternatively spliced depending on whether it is virtually included into all mature mRNAs. Because the alternatively spliced exons are not ubiquitously used, they would be less evolutionarily constrained than that of constitutively spliced exons. Therefore, we may anticipate that they would have been associated with distinct biological characteristics due to the differential evolutionary constraints. To better understand these differences will help us to explain the evolutionary origins, regulatory mechanisms, and biological implications of alternative splicing. In this paper, we focus on reviewing both sequence and evolutionary features for the alternatively spliced exons.

## 2. Alternative Splicing and Exon Classification

### 2.1. Representative Types of Alternative Splicing Events

In the classic understanding, eukaryotic genes are continually transcribed from transcriptional start to stop sites and produce pre-mRNAs, from which the noncoding introns must be precisely spliced out and coding exons are ligated to form mature mRNAs. However, the two processes of gene transcription and pre-mRNA splicing could be coupled so that splicing is also regulated by transcription factors [6,17,18]. The splicing process of pre-mRNAs is primarily carried out by the spliceosome that is a large macromolecule and composed of five small nuclear ribonucleoprotein particles (U1, U2, U4, U5 and U6 snRNPs) and hundreds of non-snRNP factors [19]. Recently, the cryo-electron microscopy structures of spliceosome have been successfully solved in human and yeast, which are very helpful to understand the accurate splicing mechanism [20,21].

**Figure 1:**
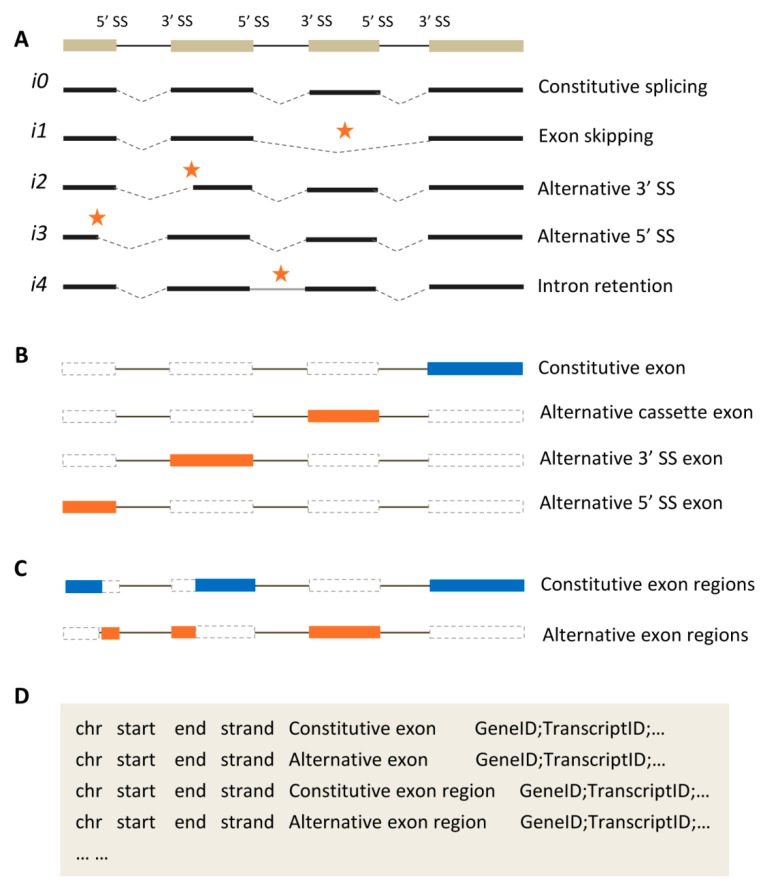
Schematic illustration of alternative splicing events and exon classification. Four representative types of alternative splicing events are demonstrated with a four-exon gene, in which each change is marked by an asterisk (**A**). The two kinds of exon classification are shown in (**B**) and (**C**), which could be accordingly annotated into a Browser Extensible Data (BED)-like file (**D**). Exons and introns are denoted by the colored boxes and solid lines, respectively. SS—splice site.

For a single pre-mRNA, multiple splice sites could be alternatively recognized and used for producing different transcript isoforms, and this process is formally defined as alternative splicing. The alternative splicing events have been generally classified into four main types at least, including the exon skipping (also called alternative cassette exon), alternative 3′ splice site (SS), alternative 5′ SS, and intron retention [22]. We have schematically illustrated a four-exon gene in Figure 1A and first shown that all exons are constitutively spliced to produce transcript *i0*. In general, transcript *i0* would have the maximum number of exons and the longest sequence in comparison with other isoforms. By comparing with *i0*, transcript *i1* represents an exon skipping in which the third exon is entirely spliced out. Similarly, alternative 3′ SS (*i2*) and 5′ SS (*i3*) events are illustrated when splice site changes on 3′ end of the second exon and 5′ end of the first exon, respectively. The second intron was not spliced out of transcript *i4* that is called intron retention. A minor type of alternative splicing event was recently suggested and named the exonic intron [23], that is the alternative inclusion for an internal region of exon (not shown). It should be noted, of course, that more than one alternative splicing event could be simultaneously observed in a single transcript isoform. In a broader sense, there are two additional types of transcript isoforms that differ on 3’ end of the first exon and 5’ end of the last exon, which are introduced at the initial and terminal stages of genic transcription, respectively.

### 2.2. Two Kinds of Exon Classification

According to the definition of alternative splicing, all exons could be classified to be either constitutively or alternatively spliced. Although this classification appears to be simple, there are some ambiguous cases when describing the different types of exons in some of the literature. For example, exons with alternative 3′/5′ SS are inconsistently treated as either constitutively or alternatively spliced exons [24,25]. Also, the term “alternatively spliced exon” would be only restricted to alternative cassette exons [26]. Therefore, here we concisely summarize the two kinds of exon classification. First, all exons could be classified into four types in accordance with the different alternative splicing events, including the constitutive exon, alternative cassette exon, alternative 3′ SS exon, and alternative 5′ SS exon (Figure 1B). Among them, the latter three types could be collectively called the alternative exon. Because this classification maintains exonic intactness, it will face an uncertainty in selecting the representative sequence among the several forms in different length for the alternative 3′/5′ SS exons. Actually, it was also suggested in some parts of the literature that both constitutive and alternative exons could be further classified into simple, multiple and complex forms according to the observed occurrence times among all transcript isoforms [25,27]. Second, a single exon is dividable and could be separately classified into the constitutive and alternative exon regions in a more simply way (Figure 1C), which means that one exon would not be treated as an intact unit. For the two kinds of exon classification, which one should be used will mainly depends on the studied biological questions. Of course, there is an ongoing debate on whether all exons would be alternatively spliced actually [25]. Also, we might keep in mind that the exon’s classification may be changeable when the different sets of transcripts are used for analysis of alternative splicing.

### 2.3. Annotating Exons

Due to the wide application of RNA-Seq, a large number of transcript sequences have been assembled and always stored in the standard general transfer format (GTF) or general feature format (GFF) files. Therefore, it is sometimes necessary to extract the constitutive and alternative exons suitable for subjecting to specific analyses, such as robust quantification of isoform expression [28]. However, it is not a trivial task for non-bioinformatic researchers to distinguish and use different types of exons. To address this issue, we prepared a bioinformatic script (available upon request) for annotating constitutive and alternative exons based on the custom set of transcripts. This script was written in the Python language and designed to separately address the two kinds of exon classification. First, each exon was maintained to be intact and directly annotated to be constitutive or alternative. Second, all exons can be divided and then classified into the constitutive and alternative exon regions. This script outputs a BED-like file that fully retains the original information from the inputted file (Figure 1D).

## 3. Sequence Features of Alternative Exons

### 3.1. Core Splicing Signals and Regulatory Elements

**Figure 2:**
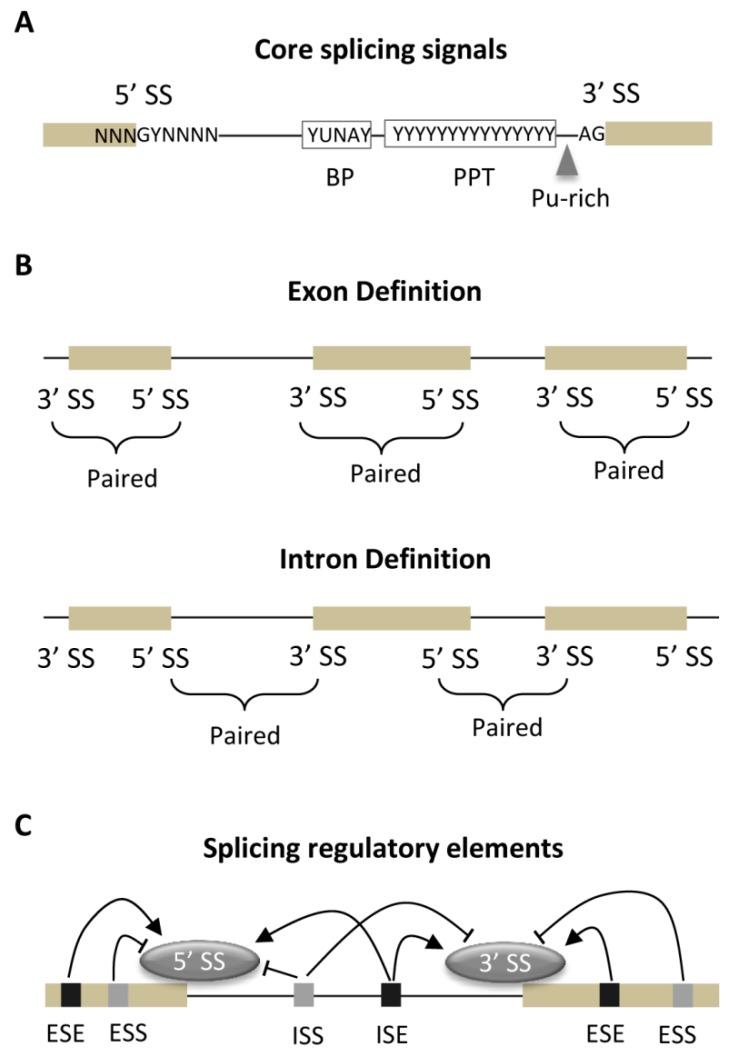
Splicing signals, recognition models and splicing regulatory elements. The core splicing signals within pre-mRNA and their consensus sequences are schematically illustrated (**A**). N, any purine or pyrimidine; Y, any pyrimidine; Pu-rich, purine-rich element. (**B**) The recognition models of spliceosome include “Exon Definition” and “Intron Definition”. The locations and roles of splicing regulatory elements are shown in (**C**), for which the three-letter abbreviations are stated in the main text. BP—branch point; PPT—polypyrimidine tract; ESS—exonic splicing silencers; ISS—intronic splicing silencers; ESE—exonic splicing enhancers; ISE—intronic splicing enhancers.

Spliceosome discriminates exonic/intronic sequences and determines the 5′ and 3′ SSs by primarily recognizing core splicing signals (Figure 2A). The 5′ SS is selected by complementary recognition between a 9-nt motif and U1 snRNP. This motif always spans the boundary between exon (−3 to −1) and intron (+1 to +6), and its nucleotide composition is the major determinant of 5′ SS selection as recently revealed by the massively parallel splicing assay [29]. For the great majority of introns, the two nucleotides at +1 and +2 positions of 5′ SS are almost conserved with GU (more than 98%) and GC (less than 1%) [30]. Although both of them have been believed to be canonical types with normal splicing, a recent study also reported that no more than 20% of GU type could retain their capacity to generate the normal transcripts when it is substituted by GC type [31]. The nucleotide composition of 5′ SS motif has been also recently updated by analyzing more than 1000 species/lineages [32].

In contrast to 5′ SS, the selection of 3′ SS is more complicated and would jointly involve three motifs that are generally located within the last region of intron with ~50 nts in length [33], including the branch point (BP), polypyrimidine tract (PPT) and AG dinucleotide at the intron/exon junction (Figure 2A). By binding to SF1/BBP, the BP is an extremely degenerate motif with consensus sequence of YUNAY in humans. Most BPs are proximally located away from 3′ SS with 14~50 nts, whereas some could be distantly located up to 350 nts [34]. The PPT is a ~20-nt pyrimidine-rich motif and adjacently located to the consensus AG dinucleotide with only 2-nt distance in general, both of which are recognized by the large subunit (U2AF65) and small subunit (U2AF35) of U2 auxiliary factor, respectively. However, a recent study also revealed that the purine-rich elements are widely inserted between the PPT and AG dinucleotide and play positive roles for regulating pre-mRNA splicing [35]. Two adjacent splice sites would be dependently recognized by spliceosome for achieving a higher precision. Therefore, the basic recognition process could be described by two models of “Exon Definition” and “Intron Definition” (Figure 2B), for which spliceosome recognizes the pairing between the two adjacent splice sites across an exon or intron, respectively [36]. The choice of recognition model would mainly depend on the relative length of exons and introns, and this exon-intron architecture is an important evolutionary feature [37].

In addition to direct contacts between spliceosome and core splicing signals, pre-mRNA splicing has been regulated by various splicing regulatory elements (SREs) that are the short motifs and enriched within both exons and introns. These SREs regulate the splicing process by recruiting the sequence-specific RNA-binding proteins (RBPs), such as the SR proteins or hnRNPs, that will either activate or inhibit the recognition and use of the adjacent splice sites [38]. Therefore, SREs have been conventionally classified as exonic/intronic splicing enhancers (ESEs/ISEs) and exonic/intronic splicing silencers (ESSs/ISSs) according to their locations and functional roles (Figure 2C). Both high-throughput computational and experimental approaches have been employed for identifying SREs. Castle et al. (2008) conducted the first genome-wide screen for 4-mer to 7-mer words and computationally identified a large number of SREs [39]. Many in silico methods for predicting SREs have been successfully developed during the past years, which was specifically reviewed recently [40]. Based on the fluorescence-based splicing reporter, the experimental approach was developed to systematically identify SREs [41]. The genome-wide discovery of SREs have been significantly advanced by combining the high-throughput sequencing with immunoprecipitation, such as the crosslinking immunoprecipitation sequencing (CLIP-seq) and RNA immunoprecipitation sequencing (RIP-seq) [42,43,44]. On the whole, hundreds of SREs have been computationally or experimentally discovered and most of them are mainly involved in regulating tissue-specific alternative splicing of pre-mRNAs [45]. Recently, it was revealed that SREs are also responsible for controlling the oscillating alternative splicing [46].

### 3.2. Strength of Core Splicing Signals

The sequence degeneracy of motifs of core splicing signals is the main basis for determining alternative splice sites to be recognized and used. Therefore, it has been widely observed that alternative exons have obvious differential sequence features in comparison with constitutive exons. Splice sites can be quantified as having strong or weak splicing strength depending on how their motifs of the core splicing signals resemble the optimal consensus sequences. In practice, a position weight matrix could be generated and used for scoring the splicing strength of 3′/5′ SS by calculating nucleotide frequencies of the motif sequences at each position [6,37]. Additionally, the physical-chemical properties, intra-motif dependencies and machine learning models have recently been successfully adopted into in silico methods for predicting the 3′/5′ SS strength [47,48,49].

In general, strong splicing strength of splice sites could facilitate unambiguous recognition by spliceosome and herein result into constitutive splicing, whereas weak splicing strength is more easily subjected to alternative splicing. Therefore, alternative cassette exons and alternative 3′/5′ SS exons have the weaker splicing strength than that of constitutive exons [50,51]. However, the relatively weaker strength of splice sites was only observed in the variable ends of alternative 3′/5′ SS exons [24]. Grau-Bové et al. (2018) comprehensively investigated the alternative splicing landscape across 65 eukaryotic species and confirmed the significant and consistent relationship between alternative cassette exon and weaker strength of both 3′ and 5′ SSs [52]. Furthermore, mutations within the recognized motifs of core splicing signals could obviously change the strength and then influence the splicing pattern. Recently, about 10% of exonic pathogenic mutations were found to actually disrupt the spliceosome assembly [53]. Jaganathan et al. (2019) employed a 32-layer deep neural network for successfully identifying the pre-mRNA splicing, which more importantly could accurately and robustly predict the effects of synonymous and intronic mutations on alternative splicing [54]. These sequence features, along with the evolutionary features that are stated below, are summarized in Table 1.

### 3.3. Distribution of SREs

Beside the core splicing signals, alternative and constitutive exons can also differ significantly on their exonic and intronic SREs. Yeo et al. (2007) first identified 314 conserved intronic SREs by comparative genomic approach and found that SREs inserted between two competitive splice sites would be much likely to generate alternative 3′/5′ SS exons [55]. By analyzing the alternative splicing landscape among 48 human tissues and cell lines, six clusters of SREs that are represented by UCUCU, UGCAUG, UGCU, UGUGU, UUUU and AGGG were found to be enriched near the alternative cassette exons, which also showed distinct patterns in terms of genomic location and tissue specificity [39]. Rosenberg et al. (2015) systematically measured the splicing patterns of synthetic mini-genes and found that the vast majority of SREs within alternative exons could influence the choice of splice sites in an additive manner [56].

The splicing enhancers would play predominant roles in constitutive splicing, while splicing silencers mainly regulate alternative splicing [38,57]. On the one hand, mutations of splicing enhancers would lead to conversion from constitutive to alternative exons. It was recently observed in human chronic granulomatous disease that the exon 5 of cytochrome b beta chain (*CYBB*) gene was skipped because of its site mutation of ESEs [58]. On the other hand, the disrupted recognition of ESE that is caused by mutations of the corresponding RBPs was also observed to induce mis-splicing of key hematopoietic regulators in myelodysplasia [59]. The splicing silencers are more abundant in the alternative exons than that in constitutive exons, and the exclusion of alternative exons frequently requires cooperative regulation by multiple silencer elements [60]. In addition, the distributed positions of SREs within exons would also have different influences on the constitutive or alternative splicing [61]. On the whole, it would be less likely to distinguish the constitutive and alternative exon-specific SREs, because most of them do actually function in a spatiotemporal regulation manner.

### 3.4. Exon-Intron Architecture

Because the exon-intron architecture significantly affects the recognition models of spliceosome, the relative length of exons and introns is an important characteristic to distinguish the constitutive and alternative exons. It has long been recognized that the alternative cassette exons are flanked by longer introns for both upstream and downstream than those of constitutive exons [52], whereas the alternative 3′/5′ SS exons don’t show such an obvious difference [62]. For example, in humans the mean length of upstream and downstream introns are ~4070 and ~3470 nts for the constitutive exons and ~5580 and ~5020 nts for the alternative cassette exons, respectively [37]. However, length differences of the flanking introns between constitutive and alternative exons were less obvious in lower vertebrates, which suggests that this feature would be a consequence of evolution [37].

Beside introns, the shorter length of alternative cassette exons was previously observed in mammals [37,63], which was recently confirmed by comprehensively analyzing 65 eukaryotic species [52]. Therefore, there is a significant association of alternative cassette exons with higher intron-to-exon length ratios. It was observed that alternative cassette exons have lower GC contents than that of constitutive exons [51]. However, the association between GC content and exon type would display species-specific differences [52]. In addition, the GC contents of exon-intron boundaries could be differentiated between constitutive and alternative exons [64]. Also, the differential GC contents between exons and introns could interact with intron length for regulating alternative splicing [65]. Furthermore, the constitutive splicing of short exons would require additional enhancers from the adjacent introns [66], which may explain why alternative cassette exons always have the shorter length.

## 4. Evolutionary Features of Alternative Exons

### 4.1. Evolutionary Ages and Inclusion Levels of Exons

**Figure 3:**
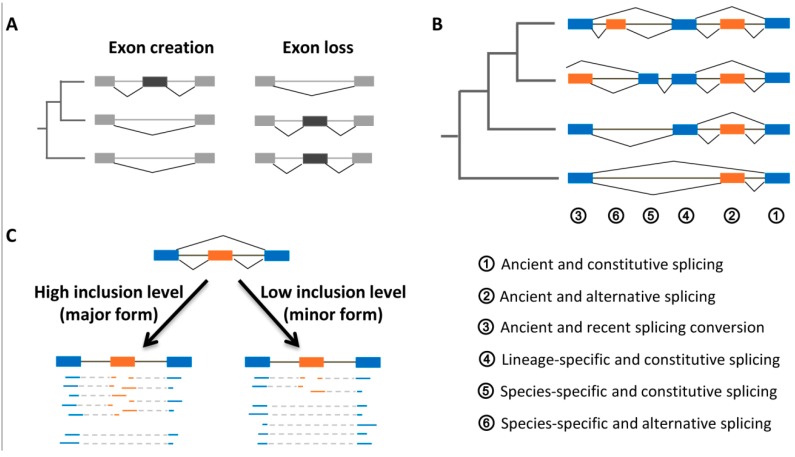
Exon evolutionary ages and inclusion levels. (**A**) Exon creation as well as loss is first illustrated within an evolutionary tree of three species. Subsequently, six exons are schematically exemplified to show the different evolutionary ages and splicing patterns (**B**). The inclusion levels of one alternative exon are detected by the spliced mapping of RNA-Seq reads against reference genome (**C**).

Within one existing gene, one or more exons can be newly created and also lost during the evolutionary process (Figure 3A). Among both of them, exon creation events are much more widespread than exon loss and hence significantly contribute to diversification of protein function [67]. New exons could derive from the external insertion/tandem duplication of existing exons and also from *de novo* exonization of intronic sequences [68]. The exonizations mainly originate from these transposable elements, such as the long interspersed nuclear elements (LINEs) and short interspersed nuclear elements (SINEs), because they always carry the consensus motifs resembling real splice sites [69]. When putting exons’ splicing patterns in an evolutionary context, they become much more complex, as shown in Figure 3B. First, exons could be assigned different evolutionary ages according to what extent they were evolutionary conserved, such as the species-specific (or recently created), lineage-specific (or early evolved), and ancient (or fully conserved) exons. Similarly, the splicing pattern of orthologous exons would change or not change when compared among different organisms. Therefore, every exon should be described by its conservation levels for both evolutionary origin and splicing pattern, for which six exons were representatively exemplified (Figure 3B).

For an alternative exon, another critical issue that should be taken into consideration is the inclusion level, which was defined as the fraction of the gene’s transcripts that include this exon [70]. In practice, the exon’s inclusion levels could be quantified by counting the mapped cDNA fragments, such as RNA-Seq reads and ESTs, in support of their respective splice junctions (Figure 3C). According to the quantified inclusion levels, an alternative exon could be subjectively classified into major and minor isoforms, both of which would have distinct and important biological implications.

### 4.2. Evolutionary Origins

The systematic investigation of exon origin and evolution was first conducted in rodents by comparative genomic analysis, which revealed that the species-specific exons are more likely to be alternatively spliced and characterized by low inclusion levels [71]. The subsequent similar studies analyzing more vertebrate species also supported an obvious relationship of an exon’s evolutionary age with both the potential of alternative splicing and inclusion levels [72,73]. However, these studies were less reliable in inferring the presence/absence of orthologous exons and estimating inclusion levels of alternative exons because they employed comparative genomic approaches and EST data. One later study comprehensively sequenced cDNA molecules among nine tissues from five vertebrates and found that the degree of evolutionary conservation for alternative splicing patterns varied substantially among different tissues, and the ancient alternative exons had the weakest strength of splice sites [74]. Furthermore, the recently converted exons from constitutive to alternative splicing had splice sites of decreased strength, whereas the inverse conversions were not associated with such changes [74]. The recent reanalysis of these RNA-Seq data further found that the species-specific alternative exons would have high inclusion levels in the specific tissue(s), which are also associated with the increased gene expression [75]. By focusing on primate lineage [76], it was suggested that changes in exon inclusion level are more likely to be functionally relevant than that of conversion of splicing pattern. Overall, these studies support the contention that alternative exons are associated with younger evolutionary ages and higher tissue-specific differences of inclusion level in comparison with constitutive exons.

The above conclusions have mainly been drawn from analyses of alternative cassette exons because they are more easily and accurately detected by comparative genomic approaches. Although it is well known that alternative 3′/5′ SS exons result from competitive usage of the cryptic splice sites, their evolutionary origins and conservation levels have not been systemically analyzed yet. A recent report studying the alternative splicing landscape in response to infection in humans suggested that the cryptic splice sites would always not be conserved [77]. Furthermore, the cryptic splice sites are generally thought to be associated with lower inclusion levels than that of the nearby canonical splice sites [78]. However, special caution should be paid to this conclusion because the inclusion levels of alternative 3’/5’ SS exons are similarly expected to be highly variable among different tissues.

### 4.3. Selective Constraints

Alternative cassette exons are the most common type of alternative splicing event in vertebrates and are thought to be less evolutionarily constrained because they are not virtually included into all mature mRNAs [79]. Higher non-synonymous substitution rates (Ka) were previously observed in alternative cassette exons than that in constitutive exons by analyzing human-mouse orthologous exons, which indicates faster evolution at the amino acid level and significant contribution to protein functional diversification [80,81]. By contrast, alternative cassette exons have lower synonymous substitution rates (Ks) and the increased conservation of nucleotide sequences [82]. Accordingly, the species-specific alternative cassette exons that originated relatively recently only have slightly increased conservation and Ka/Ks ratio [63]. In addition, the peptide sequences encoded by the alternative cassette exons are also less likely to be located within the essential structural units of proteins [83].

Alternative 3′/5′ SS exons were previously suggested to be intermediate states because the variable ends are more similar to alternative cassette exons but the fixed ends resemble constitutive exons in terms of sequence conservation level and Ka/Ks ratio [24]. Although the entire sequences of alternative 3′/5′ SS exons are less symmetrical (i.e., divisible by 3) like constitutive exons, the regions between the two competitive splice sites show high symmetry levels, and hence are more similar to alternative cassette exons [24,63]. High symmetry levels were also observed for the alternatively spliced internal regions of protein-coding exons [23]. A recent study further revealed that non-synonymous mutations were preferentially located within the alternatively spliced coding regions specific to the minor transcript isoforms [84]. All of these results support the supposition that there are different evolutionary constraints between the constitutive and alternative exons.

### 4.4. Regulatory and Coding Roles

Alternative splicing events have been found to not be evenly distributed throughout the mRNA molecules and are hence differentially involved in regulatory and coding roles. The alternative exons, especially for these evolutionary young exons [72], are more likely to lie within or adjacent to the untranslated regions (UTRs), which is also consistent with the recent observation in humans that the alternative splicing of UTRs was very common and often highly complex [7]. Therefore, the inclusion or exclusion of alternative exons within 5′ and 3′ UTRs would positively play regulatory roles by influencing the mRNA translational efficiency, second structure, stability, and subcellular localization, which was recently reviewed [85]. However, the more ancient alternative exons are more likely located within coding regions for producing distinct protein isoforms [74]. In addition to coding the additional amino acid segments, inclusion of alternative exons could also provide preferable translation start/end sites that would yield the truncated proteins [86]. These differences indicate the important consequences of evolutionary regulation, because a large proportion of species- and lineage-specific alternative exons are restrictively expressed in the specific tissues and developmental stages.

## 5. Conclusions

Here, we provide an overview of several issues in relation to alternative splicing of eukaryotic genes, mainly focusing on sequence and evolutionary features for the alternatively spliced exons. Nevertheless, some interesting topics still remain to be specially addressed in the future, such as bioinformatic approaches for identifying allele-specific alternative splicing events from RNA-Seq data.

## Figures and Tables

**Table 1:** Summary of sequence and evolutionary features.

	Items	Summary Description
**Sequence features**	Core splicing signals	▪ Splice sites could be quantified into the strong or weak splicing strength according to their motif sequences. ▪ Alternative exons have the weaker strength at variable end(s). ▪ Mutations within motifs could affect the splicing pattern.
	Splicing regulatory elements (SREs)	▪ Splicing enhancers and silencers play the predominant roles for determining constitutive and alternative splicing, respectively. ▪ Distributed density and positions of SREs could affect the splicing pattern. ▪ Multiple SREs would function in an additive manner. ▪ Mutations within SREs could affect the splicing pattern.
	Exon-intron architecture	▪ Alternative cassette exons are shorter and flanked by the longer introns, which leads to higher intron-to-exon length ratios. ▪ Constitutive and alternative exons have differential GC contents at the exon-intron boundaries. ▪ Constitutive exons in short length require additional splicing enhancers from the adjacent introns.
**Evolutionary features**	Origin	▪ The evolutionary young exons are more likely to be alternatively spliced and have the high inclusion levels only in specific tissue(s). ▪ Evolutionary conversion from constitutive to alternative exons is associated with the decreased splicing strength. ▪ Changes in exon inclusion level are more likely to be functionally relevant.
	Selective constraint	▪ Alternative cassette exons have the faster evolution at amino acid level and higher conservation of nucleotide sequence. ▪ Alternative 3′/5′ SS exons have differential selective constraints between the variable and fixed ends. ▪ Alternative 3′/5′ SS exons have high symmetry levels for the alternative region between two competitive splice sites.
	Regulatory and coding roles	▪ The evolutionary young exons are more likely located within UTRs and play the regulatory roles. ▪ Ancient alternative exons are more likely involved in producing the distinct protein isoforms.

## References

[1] Kelly D.E., Hansen M.E.B., Tishkoff S.A. (2017). Global variation in gene expression and the value of diverse sampling. Curr. Opin. Syst. Biol..

[2] Portin P., Wilkins A. (2017). The evolving definition of the term “gene”. Genetics.

[3] Wong C.-M., Xu L., Yau M.Y.-C. (2018). Alternative mRNA splicing in the pathogenesis of obesity. Int. J. Mol. Sci..

[4] Zhang Y., Wang X., Jiang Q., Hao H., Ju Z., Yang C., Sun Y., Wang C., Zhong J., Huang J. (2018). DNA methylation rather than single nucleotide polymorphisms regulates the production of an aberrant splice variant of *IL6R* in mastitic cows. Cell Stress Chaperones.

[5] Shang X., Cao Y., Ma L. (2017). Alternative splicing in plant genes: A means of regulating the environmental fitness of plants. Int. J. Mol. Sci..

[6] Kornblihtt A.R., Schor I.E., Alló M., Dujardin G., Petrillo E., Muñoz M.J. (2013). Alternative splicing: A pivotal step between eukaryotic transcription and translation. Nat. Rev. Mol. Cell Biol..

[7] Deveson I.W., Brunck M.E., Blackburn J., Tseng E., Hon T., Clark T.A., Clark M.B., Crawford J., Dinger M.E., Nielsen L.K. (2018). Universal alternative splicing of noncoding exons. Cell. Syst..

[8] Park E., Pan Z., Zhang Z., Lin L., Xing Y. (2018). The expanding landscape of alternative splicing variation in human populations. Am. J. Hum. Genet..

[9] Brett D., Pospisil H., Valcárcel J., Reich J., Bork P. (2001). Alternative splicing and genome complexity. Nat. Genet..

[10] Pan Q., Shai O., Lee L.J., Frey B.J., Blencowe B.J. (2008). Deep surveying of alternative splicing complexity in the human transcriptome by high-throughput sequencing. Nat. Genet..

[11] Wang E.T., Sandberg R., Luo S., Khrebtukova I., Zhang L., Mayr C., Kingsmore S.F., Schroth G.P., Burge C.B. (2008). Alternative isoform regulation in human tissue transcriptomes. Nature.

[12] Tress M.L., Abascal F., Valencia A. (2017). Alternative splicing may not be the key to proteome complexity. Trends Biochem. Sci..

[13] Kahles A., Ong C.S., Zhong Y., Rätsch G. (2016). SplAdder: Identification, quantification and testing of alternative splicing events from RNA-Seq data. Bioinformatics.

[14] Ji G., Ye W., Su Y., Chen M., Huang G., Wu X. (2018). AStrap: Identification of alternative splicing from transcript sequences without a reference genome. Bioinformatics.

[15] Weirather J.L., de Cesare M., Wang Y., Piazza P., Sebastiano V., Wang X.-J., Buck D., Au K.F. (2017). Comprehensive comparison of Pacific Biosciences and Oxford Nanopore Technologies and their applications to transcriptome analysis. F1000Res..

[16] Chen S.-Y., Deng F., Jia X., Li C., Lai S.-J. (2017). A transcriptome atlas of rabbit revealed by PacBio single-molecule long-read sequencing. Sci. Rep..

[17] Naftelberg S., Schor I.E., Ast G., Kornblihtt A.R. (2015). Regulation of alternative splicing through coupling with transcription and chromatin structure. Annu. Rev. Biochem..

[18] Lee Y.J., Wang Q., Rio D.C. (2018). Coordinate regulation of alternative pre-mRNA splicing events by the human RNA chaperone proteins hnRNPA1 and DDX5. Genes Dev..

[19] Papasaikas P., Valcárcel J. (2016). The spliceosome: The ultimate RNA chaperone and sculptor. Trends Biochem. Sci..

[20] Zhang X., Yan C., Hang J., Finci L.I., Lei J., Shi Y. (2017). An atomic structure of the human spliceosome. Cell.

[21] Bai R., Wan R., Yan C., Lei J., Shi Y. (2018). Structures of the fully assembled *Saccharomyces cerevisiae* spliceosome before activation. Science.

[22] Black D.L. (2003). Mechanisms of alternative pre-messenger RNA splicing. Annu. Rev. Biochem..

[23] Marquez Y., Höpfler M., Ayatollahi Z., Barta A., Kalyna M. (2015). Unmasking alternative splicing inside protein-coding exons defines exitrons and their role in proteome plasticity. Genome Res..

[24] Koren E., Lev-Maor G., Ast G. (2007). The emergence of alternative 3′ and 5′ splice site exons from constitutive exons. PLoS Comput. Biol..

[25] Chen F.-C. (2013). Are all of the human exons alternatively spliced?. Brief. Bioinform..

[26] Dror G., Sorek R., Shamir R. (2004). Accurate identification of alternatively spliced exons using support vector machine. Bioinformatics.

[27] Song S., Huang Q., Guo J., Li-Ling J., Chen X., Ma F. (2009). Comparative component analysis of exons with different splicing frequencies. PLoS ONE.

[28] Han Y., Gao S., Muegge K., Zhang W., Zhou B. (2015). Advanced applications of RNA sequencing and challenges. Bioinform. Biol. Insights.

[29] Wong M.S., Kinney J.B., Krainer A.R. (2018). Quantitative activity profile and context dependence of all human 5′ splice sites. Mol. Cell.

[30] Sheth N., Roca X., Hastings M.L., Roeder T., Krainer A.R., Sachidanandam R. (2006). Comprehensive splice-site analysis using comparative genomics. Nucleic Acids Res..

[31] Lin J.-H., Tang X.-Y., Boulling A., Zou W.-B., Masson E., Fichou Y., Raud L., Le Tertre M., Deng S.-J., Berlivet I. (2019). First estimate of the scale of canonical 5′ splice site GT>GC variants capable of generating wild-type transcripts. Hum. Mutat..

[32] Nguyen H., Das U., Wang B., Xie J. (2018). The matrices and constraints of GT/AG splice sites of more than 1000 species/lineages. Gene.

[33] Von Voithenberg L.V., Sánchez-Rico C., Kang H.-S., Madl T., Zanier K., Barth A., Warner L.R., Sattler M., Lamb D.C. (2016). Recognition of the 3′ splice site RNA by the U2AF heterodimer involves a dynamic population shift. Proc. Natl. Acad. Sci. USA.

[34] Wen J., Wang J., Zhang Q., Guo D. (2017). A heuristic model for computational prediction of human branch point sequence. BMC Bioinform..

[35] Nguyen H., Xie J. (2019). Widespread separation of the polypyrimidine tract from 3′ AG by G tracts in association with alternative exons in Metazoa and Plants. Front. Genet..

[36] De Conti L., Baralle M., Buratti E. (2013). Exon and intron definition in pre-mRNA splicing. Wiley Interdiscip. Rev. RNA.

[37] Gelfman S., Burstein D., Penn O., Savchenko A., Amit M., Schwartz S., Pupko T., Ast G. (2012). Changes in exon-intron structure during vertebrate evolution affect the splicing pattern of exons. Genome Res..

[38] Wang Z., Burge C.B. (2008). Splicing regulation: From a parts list of regulatory elements to an integrated splicing code. RNA.

[39] Castle J.C., Zhang C., Shah J.K., Kulkarni A.V., Kalsotra A., Cooper T.A., Johnson J.M. (2008). Expression of 24,426 human alternative splicing events and predicted *cis* regulation in 48 tissues and cell lines. Nat. Genet..

[40] Grodecká L., Buratti E., Freiberger T. (2017). Mutations of pre-mRNA splicing regulatory elements: Are predictions moving forward to clinical diagnostics?. Int. J. Mol. Sci..

[41] Wang Y., Wang Z. (2014). Systematical identification of splicing regulatory *cis*-elements and cognate *trans*-factors. Methods.

[42] Zhang Z., Xing Y. (2017). CLIP-seq analysis of multi-mapped reads discovers novel functional RNA regulatory sites in the human transcriptome. Nucleic Acids Res..

[43] Ji X., Humenik J., Liebhaber S.A. (2019). A cytosine-rich splice-regulatory determinant enforces functional processing of the human *α-globin* gene transcript. Blood.

[44] Licatalosi D.D., Mele A., Fak J.J., Ule J., Kayikci M., Chi S.W., Clark T.A., Schweitzer A.C., Blume J.E., Wang X. (2008). HITS-CLIP yields genome-wide insights into brain alternative RNA processing. Nature.

[45] Badr E., ElHefnawi M., Heath L.S. (2016). Computational identification of tissue-specific splicing regulatory elements in human genes from RNA-Seq data. PLoS ONE.

[46] Goldammer G., Neumann A., Strauch M., Müller-McNicoll M., Heyd F., Preußner M. (2018). Characterization of *cis*-acting elements that control oscillating alternative splicing. RNA Biol..

[47] Tang R., Prosser D.O., Love D.R. (2016). Evaluation of bioinformatic programmes for the analysis of variants within splice site consensus regions. Adv. Bioinform..

[48] Xu Z.-C., Wang P., Qiu W.-R., Xiao X. (2017). iSS-PC: Identifying splicing sites via physical-chemical properties using deep sparse auto-encoder. Sci. Rep..

[49] Bretschneider H., Gandhi S., Deshwar A.G., Zuberi K., Frey B.J. (2018). COSSMO: Predicting competitive alternative splice site selection using deep learning. Bioinformatics.

[50] Busch A., Hertel K.J. (2015). Splicing predictions reliably classify different types of alternative splicing. RNA.

[51] Cui Y., Cai M., Stanley H.E. (2017). Comparative analysis and classification of cassette exons and constitutive exons. Biomed Res. Int..

[52] Grau-Bové X., Ruiz-Trillo I., Irimia M. (2018). Origin of exon skipping-rich transcriptomes in animals driven by evolution of gene architecture. Genome Biol..

[53] Soemedi R., Cygan K.J., Rhine C.L., Wang J., Bulacan C., Yang J., Bayrak-Toydemir P., McDonald J., Fairbrother W.G. (2017). Pathogenic variants that alter protein code often disrupt splicing. Nat. Genet..

[54] Jaganathan K., Panagiotopoulou S.K., McRae J.F., Darbandi S.F., Knowles D., Li Y.I., Kosmicki J.A., Arbelaez J., Cui W., Schwartz G.B. (2019). Predicting splicing from primary sequence with deep learning. Cell.

[55] Yeo G.W., Van Nostrand E.L., Liang T.Y. (2007). Discovery and analysis of evolutionarily conserved intronic splicing regulatory elements. PLoS Genet..

[56] Rosenberg A.B., Patwardhan R.P., Shendure J., Seelig G. (2015). Learning the sequence determinants of alternative splicing from millions of random sequences. Cell.

[57] Barash Y., Calarco J.A., Gao W., Pan Q., Wang X., Shai O., Blencowe B.J., Frey B.J. (2010). Deciphering the splicing code. Nature.

[58] De Boer M., van Leeuwen K., Geissler J., Belohradsky B.H., Kuijpers T.W., Roos D. (2017). Mutation in an exonic splicing enhancer site causing chronic granulomatous disease. Blood Cells Mol. Dis..

[59] Kim E., Ilagan J.O., Liang Y., Daubner G.M., Lee S.C.-W., Ramakrishnan A., Li Y., Chung Y.R., Micol J.-B., Murphy M.E. (2015). SRSF2 mutations contribute to myelodysplasia by mutant-specific effects on exon recognition. Cancer Cell.

[60] Jin Y., Dong H., Shi Y., Bian L. (2018). Mutually exclusive alternative splicing of pre-mRNAs. Wiley Interdiscip. Rev. RNA.

[61] Anczuków O., Akerman M., Cléry A., Wu J., Shen C., Shirole N.H., Raimer A., Sun S., Jensen M.A., Hua Y. (2015). SRSF1-regulated alternative splicing in breast cancer. Mol. Cell.

[62] Kim E., Magen A., Ast G. (2006). Different levels of alternative splicing among eukaryotes. Nucleic Acids Res..

[63] Lev-Maor G., Goren A., Sela N., Kim E., Keren H., Doron-Faigenboim A., Leibman-Barak S., Pupko T., Ast G. (2007). The “alternative” choice of constitutive exons throughout evolution. PLoS Genet..

[64] Baralle F.E., Giudice J. (2017). Alternative splicing as a regulator of development and tissue identity. Nat. Rev. Mol. Cell Biol..

[65] Amit M., Donyo M., Hollander D., Goren A., Kim E., Gelfman S., Lev-Maor G., Burstein D., Schwartz S., Postolsky B. (2012). Differential GC content between exons and introns establishes distinct strategies of splice-site recognition. Cell Rep..

[66] Li Y.I., Sanchez-Pulido L., Haerty W., Ponting C.P. (2015). RBFOX and PTBP1 proteins regulate the alternative splicing of micro-exons in human brain transcripts. Genome Res..

[67] Wang J., Lu Z.-X., Tokheim C.J., Miller S.E., Xing Y. (2014). Species-specific exon loss in human transcriptomes. Mol. Biol. Evol..

[68] Keren H., Lev-Maor G., Ast G. (2010). Alternative splicing and evolution: Diversification, exon definition and function. Nat. Rev. Genet..

[69] Bourque G., Burns K.H., Gehring M., Gorbunova V., Seluanov A., Hammell M., Imbeault M., Izsvák Z., Levin H.L., Macfarlan T.S. (2018). Ten things you should know about transposable elements. Genome Biol..

[70] Modrek B., Lee C.J. (2003). Alternative splicing in the human, mouse and rat genomes is associated with an increased frequency of exon creation and/or loss. Nat. Genet..

[71] Wang W., Zheng H., Yang S., Yu H., Li J., Jiang H., Su J., Yang L., Zhang J., McDermott J. (2005). Origin and evolution of new exons in rodents. Genome Res..

[72] Zhang X.H.-F., Chasin L.A. (2006). Comparison of multiple vertebrate genomes reveals the birth and evolution of human exons. Proc. Natl. Acad. Sci. USA.

[73] Alekseyenko A.V., Kim N., Lee C.J. (2007). Global analysis of exon creation versus loss and the role of alternative splicing in 17 vertebrate genomes. RNA.

[74] Merkin J., Russell C., Chen P., Burge C.B. (2012). Evolutionary dynamics of gene and isoform regulation in Mammalian tissues. Science.

[75] Merkin J.J., Chen P., Alexis M.S., Hautaniemi S.K., Burge C.B. (2015). Origins and impacts of new mammalian exons. Cell Rep..

[76] Xiong J., Jiang X., Ditsiou A., Gao Y., Sun J., Lowenstein E.D., Huang S., Khaitovich P. (2018). Predominant patterns of splicing evolution on human, chimpanzee and macaque evolutionary lineages. Hum. Mol. Genet..

[77] Rotival M., Quach H., Quintana-Murci L. (2019). Defining the genetic and evolutionary architecture of alternative splicing in response to infection. Nat. Commun..

[78] DeBoever C., Ghia E.M., Shepard P.J., Rassenti L., Barrett C.L., Jepsen K., Jamieson C.H., Carson D., Kipps T.J., Frazer K.A. (2015). Transcriptome sequencing reveals potential mechanism of cryptic 3’ splice site selection in *SF3B1*-mutated cancers. PLoS Comput. Biol..

[79] Hyung D., Kim J., Cho S.Y., Park C. (2017). ASpedia: A comprehensive encyclopedia of human alternative splicing. Nucleic Acids Res..

[80] Chen F.-C., Wang S.-S., Chen C.-J., Li W.-H., Chuang T.-J. (2006). Alternatively and constitutively spliced exons are subject to different evolutionary forces. Mol. Biol. Evol..

[81] Chen F.-C., Liao B.-Y., Pan C.-L., Lin H.-Y., Chang A.Y.-F. (2012). Assessing determinants of exonic evolutionary rates in mammals. Mol. Biol. Evol..

[82] Plass M., Eyras E. (2006). Differentiated evolutionary rates in alternative exons and the implications for splicing regulation. BMC Evol. Biol..

[83] Gelly J.-C., Lin H.-Y., de Brevern A.G., Chuang T.-J., Chen F.-C. (2012). Selective constraint on human pre-mRNA splicing by protein structural properties. Genome Biol. Evol..

[84] Liu T., Lin K. (2015). The distribution pattern of genetic variation in the transcript isoforms of the alternatively spliced protein-coding genes in the human genome. Mol. Biosyst..

[85] Mockenhaupt S., Makeyev E.V. (2015). Non-coding functions of alternative pre-mRNA splicing in development. Semin. Cell Dev. Biol..

[86] Blechingberg J., Poulsen A.S.A., Kjølby M., Monti G., Allen M., Ivarsen A.K., Lincoln S.J., Thotakura G., Vægter C.B., Ertekin-Taner N. (2018). An alternative transcript of the Alzheimer’s disease risk gene *SORL1* encodes a truncated receptor. Neurobiol. Aging.

